# High prevalence of anterior pituitary deficiencies after cranial radiation therapy for skull base meningiomas

**DOI:** 10.1186/s12885-021-09045-3

**Published:** 2021-12-18

**Authors:** Perrine Raymond, Marc Klein, Thomas Cuny, Olivier Klein, Julia Salleron, Valérie Bernier-Chastagner

**Affiliations:** 1 Department of radiation therapy, Institut de Cancérologie de Lorraine, 6 avenue de Bourgogne, 54519 Vandoeuvre Les Nancy, France; 2 Department of Endocrinology, University hospital CHU de Nancy, Rue du Morvan, 54500 Vandoeuvre Les Nancy, France; 3 Department of Endocrinology, Hôpital de la Conception, Aix Marseille Univ, APHM, Inserm, MMG, Marseille, France; 4 Department of Neurosurgery, University hospital CHU de Nancy, Nancy, France; 5 Department of biostatistics, Institut de Cancérologie de Lorraine, Université de Lorraine F-54519, 6 avenue de Bourgogne, 54519 Vandoeuvre Les Nancy, France; 6 Department of radiation therapy, Institut de Cancérologie de Lorraine, 6 avenue de Bourgogne, 54519 Vandoeuvre Les Nancy, France

**Keywords:** Skull base meningiomas, Radiation therapy, Pituitary deficiencies, Increase morbi-mortality

## Abstract

**Background:**

Cranial irradiation represents one of the first line treatment proposed in skull base meningiomas. While cranial irradiation is associated with a high risk of secondary hypopituitarism, few studies focused on the specific location of skull base meningiomas.

**Methods:**

Fifty-two adults receiving photon-beam therapy for skull base meningiomas between 2003 and 2014 in our Institution were included. Anterior pituitary (ACTH, FSH, GH, LH, TSH and prolactin) as well as corresponding peripheral hormones (8 am-Cortisol, IGF-1, fT3, fT4, 17βestradiol or testosterone) were biologically screened before radiotherapy (baseline), then yearly until March 2019. The pituitary gland (PG) was delineated on CT and the mean dose delivered to it was calculated.

**Results:**

Mean age at diagnosis was 56 +/− 14 years. Median follow-up was 7 years. Up to 60% of patients developed at least ≥2 pituitary deficiencies, 10 years after radiotherapy. Gonadotroph, thyrotroph, corticotroph and somatotroph deficiencies occurred in 37, 28, 18 and 15% of patients, respectively. Hyperprolactinemia was found in 13% of patients. None patient had only one pituitary deficiency. In the multivariate analysis, a delivered dose to the PG ≥ 50 Gy or a meningioma size ≥40 mm significantly increased the risk of developing hypopituitarism.

**Conclusions:**

Over a long-term follow-up, cranial radiation therapy used in skull base meningiomas led to a high prevalence of hypopituitarism, further pronounced in case of tumor ≥4 cm. These results advocate for an annual and prolonged follow-up of the pituitary functions in patients with irradiated skull base meningiomas.

## Background

Meningiomas are the most common benign intracranial tumors, with an incidence of 8.3 per 100.000 population between 2003 and 2014, representing 36.6% of primary cerebral tumors [1]. Surgery is recommended as the first-line treatment in most cases with high rate of progression-free survival ranging from 96% at 3 years to 93% at 5 years [2, 3]. According to specific guidelines, radiation therapy can also be proposed in first line treatment if the lesion is inoperable, if symptoms do appears or if the lesion progress during a follow-up [4]. When meningiomas are located near to the nerves or blood vessels, like in the skull base, surgery is more limited and exposes the patient to a high risk of recurrence [5]. In such cases, irradiation can be proposed, however few datas focused on the potential onset of radiotherapy-induced pituitary deficiencies. If untreated, the latter have a negative impact on quality of life [6, 7]. Hypopituitarism has been shown to increase morbi-mortality, and more specifically in young women with untreated gonadotroph deficiency [8, 9], and at-risk patients therefore require a regular follow-up to both diagnose and treat the outcome of hormonal deficiency (ies). In our study, our aim was to retrospectively assess the rates of anterior pituitary deficiencies in a population of patients treated by cranial radiation therapy for a skull base meningioma.

## Methods

We retrospectively reviewed the records of patients treated between 2003 and 2014 in our institution (Institut de Cancérologie de Lorraine) for skull base meningiomas, anterior or medial localization, (either confirmed histologically or presumed) by cerebral radiation therapy involving the pituitary gland within the radiation field.

All included patients were ≥ 18 years of age with normal values for the 5 anterior pituitary axis upon neuroendocrine analysis before the initiation of radiotherapy protocol.

### Endocrinology

Before the first cranial irradiation session, patients underwent an initial hormonal analysis, which was not necessarily performed in our center. This hormonal check-up was considered as the baseline and comprised anterior pituitary hormones (ACTH, FSH, GH, LH, TSH and prolactin) as well as corresponding peripheral hormones (8 am-Cortisol, IGF-1, fT3, fT4, 17bestradiol or testosterone).

Neuroendocrine analyses tested all five of the axes involving the anterior pituitary gland: the corticotroph (ACTH and 8 am-Cortisol), somatotroph (IGF-1 and GH), gonadotroph (LH, FSH, 17bestradiol or testosterone), thyrotroph (TSH, fT3, fT4), and lactotroph axis (Prolactin). The diagnosis of Hypopituitarism was considered when a deficiency of ≥1 of the 5 axis was found and panhypopituitarism when all 5 axes were deficient. A dysfunction of the lactotroph axis was suspected on hyperprolactinemia.

Dysfunction were determined by comparison to normal values for each tested hormone or marker as follows:

For the corticotroph axis, adrenal insufficiency was based on low cortisol (less than 50 mcg/L or 138 nmol/L) with an inappropriate (low or normal) ACTH levels.

For the somatotroph axis, a low IGF1 in the absence of renal insufficiency, malnutrition, hepatic dysfunction and unequilibrated insulin production was considered as GH deficiency. No dynamic test was performed and a low GH was not considered as GH deficiency due to the pulsatile nature of GH secretion. Nevertheless, a low GH alongside an inappropriate low IGF1 was considered at risk of GH deficiency.

For the gonadotroph axis, follicle-stimulating (FSH) and luteinizing (LH) hormones as well as testosterone for men and estradiol for women were analyzed. For men, low testosterone with an inappropriate FSH or LH was considered as evidence of hypogonadism. For women, an inappropriately low or normal FSH with low estradiol was considered evidence of gonadotropin dysfunction (particularly in menopausal women).

For the thyrotroph axis, low T4L or T3L with inappropriately low or normal TSH, according to the assay reference range, was considered evidence of central hypothyroidism.

Finally, for the lactotroph axis, a very low level of prolactin (below the normal range) was in favor of a lactotroph deficiency, while hyperprolactinemia could be interpretated as the reflect of an hypothalamic dysfunction by lesion of dopaminergic neurons.

### Radiation technique

All patients underwent radiation using photon-beam therapy either as first-line treatment or following initial surgery (partial or total), with fractionated daily doses of 1.8 to 2 Gy, giving a total dose of 54 to 60 Gy (mean 54.1 Gy +/− 1.6 min 50 Gy max 60 Gy). A cranial thermomask was fitted and a radiotherapy-planning computed tomography scan performed for all patients.

The gross tumor volume (GTV) was defined as the radiographically visible tumor, usually with dural extension, or as the post-operative cavity. Clinical target volume (CTV) was defined with a margin of 1 cm around the GTV, adapted to the cerebral anatomy. A margin of 0.3 cm was added to obtain planning target volume (PTV).

Constraints at delineated organs-at-risk were respected, and the pituitary region was also delineated to minimize radiation-induced toxicity. During the treatment, patients underwent weekly consultations with a radiation oncologist to detect any early side effects. After treatment, patients were followed up once every 6 months during the first 2 years and then once annually, consisting of an MRI and complete neuroendocrine analysis.

### Statistical analysis

Quantitative parameters were described as the mean and standard deviation or as median and interquartile range according to the normality of the distribution, as assessed by the Shapiro-Wilk test. Qualitative parameters were expressed as frequency and percentage.

Cumulative incidence of each deficiency was described with the Kaplan Meier method and was expressed with 95% confidence interval. Prognostic factors of at least one anterior pituitary deficiency were investigated by the Cox proportional hazard model in bivariate analyses. Quantitative variables were transformed into binary variables. The threshold corresponded to the value that maximized the Wald statistic value in the bivariate Cox model. Parameters with a *p*-value less than 0.05 were introduced in a multivariate model with backward selection. Results were expressed as hazard ratios and their 95% confidence intervals.

Statistical analysis was performed using SAS software, version 9 (SAS Institute Inc., Cary, NC). The threshold for statistical significance was set to *p* < 0.05.

## Results

Out of 200 meningiomas screened between 2003 and 2014, 83 were not located at the skull base. A total of 117 skull base meningiomas were then analyzed and 65 of them were excluded to the final analysis because of an age at diagnosis < 18 years old, a previous undergone radiosurgery, no or abnormal results from neuroendocrine analysis before radiotherapy initiation, a current hormone replacement therapy (like birth estroprogestative pill or menopausal treatment), or a chronic exposure to glucocorticoids).

**Fig. 1:**
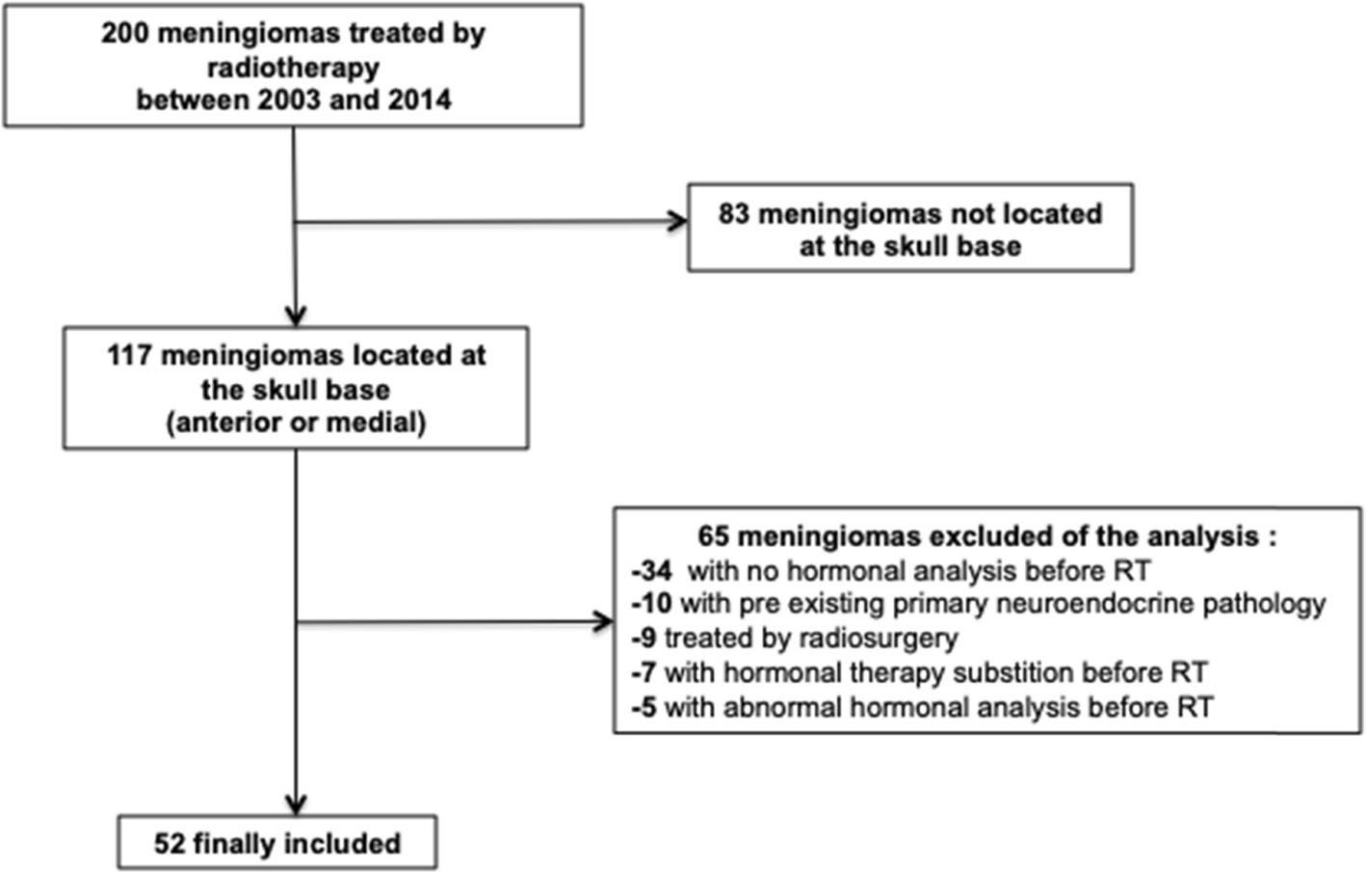
Flow chart of patient inclusion and exclusion in the study. Abbreviation: RT: Radiotherapy

A total of 52 patients with skull base meningioma who underwent cranial radiation therapy and subsequently neuroendocrine analysis follow-up were included (Flow chart, Fig. 1) including 21 anterior skull base meningiomas on the sphenoidal bone and 31 medial skull base localization (20 located at the cavernous sinus, 7 at the cerebellopontine angle, 4 petro clival).

The patient characteristics are listed in Table 1**.**

**Table 1:** Characteristics of the 52 analyzed patients

Factor	Value
Total number of patients	52
Age at diagnosis (years)	56.2 +/− 14.1
Sex	
Female	42 (80.8%)
Male	10 (19.2%)
Post menopause women	26 (50%)
BMI (kg/m2)	
< 18,5	3 (5.8%)
18,5–24,9	38 (73%)
> 25	6 (11.5%)
> 30	5 (9.7%)
Symptomatic disease manifestation	51 (98.1%)
Localization of skull base meningiomas:	
Anterior	21/52 (40.4%)
Medial	31/52 (59.6%)
Surgical resection before radiotherapy:	18 (34.6%)
Gross total resection	3 (16.7%)
Subtotal resection	14 (77.8%)
Biopsy	1 (5.5%)
WHO tumor grade	
I	17 (32.7%)
II	1 (1.9%)
Size of meningioma (mm)	27.8 +/− 13.9
Radiation therapy characteristics	
IMRT	52 (100%)
Energy	
10X	10 (19.2%)
6X	42 (80.8%)
Overall treatment time (days)	42.3 +/− 5.3
Number of fractions	28.5 +/− 1.8
Total dose (Gy)	54.1 +/− 1.6
Dose to HP axis (Gy)	47 +/− 9.4

Results are presented as mean+/− standard deviation or by frequency and percentage.

*Abbreviations*: *BMI* Body Mass Index, *WHO* World Health Organization, *IMRT* Intensity Modulated Radiation Therapy, *Gy* Gray

The median follow-up time was 7 years with an interquartile range of 5 to 10 years and 25% of patients had a follow-up greater than 10 years.

At least 2 pituitary deficiencies were present in 60.1% patients at 10 years, 95% confidence interval [40.8%; 80.1%]. At last follow-up, a total of 22 patients presented a pituitary deficiency as follows: 2 axis affected (*n* = 11), 3 axis affected (*n* = 4), 4 axis were affected (*n* = 3). A total of 4 patients had panhypopituitarism. No posterior pituitary deficiency was identified during the follow up.

**Fig. 2:**
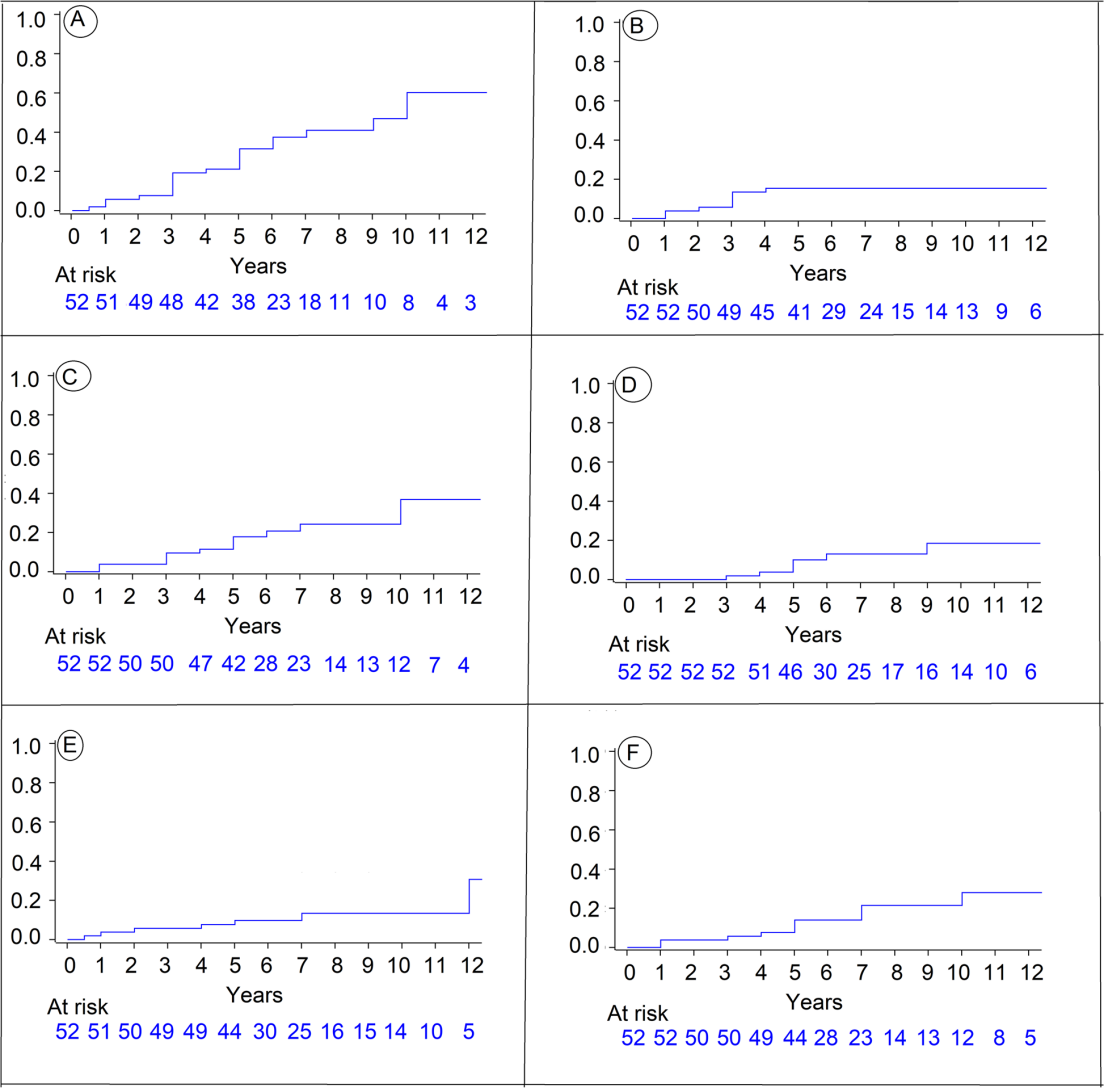
Incidence of pituitary deficiency. **A**:At least one of the 5 axes. **B** The corticotroph axis, **C** The gonadotroph axis. **D** The lactotroph axis. **E** The somatotroph axis. **F** The thyrotroph axis

The incidence of hypopituitarism and deficiency for each of the 5 axes during the follow-up period is shown in Fig. 2**(**graphic panel, Fig. 2).

At 10 years follow-up, the gonadotroph axis was the most affected with 36.9% [21.1%; 59.1%], followed by the thyrotroph axis (28.0%[14.6%; 49.4%]), the corticotroph axis (15.4%[6.7%; 28.4%]) and finally the least affected was the somatotroph axis (13.4%[6%; 28.3%]).

Hyperprolactinemia appears in 18.5%[8.5%; 37.6%], without any real deficiency (no low prolactin was assessed). 2.3% of the patients with gonadotroph deficiency had also hyperprolactinemia.

Pituitary deficiency appeared at different timeline within the first years of follow-up with a progressive increase of incidence at 3 and 5 years. Indeed, at 3 years follow up; the corticotroph axis was the most affected with 13.5% at 3 years, 15.4% at 5 years and stagnation at 15.4% at 10 years.

The results are summarized in Table 2.

**Table 2:** Anterior pituitary dysfunction after cranial irradiation for skull base meningioma

	Follow-up (years)		
Hormonal axis dysfunction n (%)	3	5	10
At least one of the 5 axes	10/52 (19.2%)	16/39 (31.5%)	22/26 (60.2%)
Corticotroph	7/52 (13.5%)	8/37 (15.4%)	8/17 (15.4%)
Somatotroph	3/52 (5.8%)	5/35 (9.8%)	6/16 (13.4%)
Gonadotroph	5/52 (9.6%)	9/37 (17.8%)	13/20 (36.9%)
Thyrotroph	3/52 (5.8%)	7/35 (14%)	10/18 (28%)
Lactotroph	1/52 (1.9%)	5/35 (10.1%)	7/17 (18.5%)

Prognostic factors of at least one anterior pituitary deficiency are presented **in** Table 3**.**

**Table 3:** Prognostic factors of at least one anterior pituitary deficiency

	Univariate analysis		Multivariate analysis	
	Hazard ratio and 95% confidence interval	*P*-value	Hazard ratio and 95% confidence interval	*P*-value
Size of tumor (mm)				
≤40	1		1	
> 40	2.98 [1.03;8.60]	0.044	4.01 [1.32;12.16]	0.014
Mean dose to pituitary (Gy)				
< 50	1		1	
≥ 50	3.63 [1.07;12.30]	0.039	2.68 [1.11;6.44]	0.028
Age at diagnosis (years)	0.99 [0.96;1.03]	0.741		
Sex				
Male	1			
Female	0.86 [0.29;2.56]	0.790		
Surgery				
No	1			
Yes	1.64 [0.70;3.84]	0.251		

In univariate analyses, the risk of hypopituitarism significantly increased when the size of tumor exceeded 40 mm (HR 2.98, 95% CI [1.03; 8.60]) and when the mean dose delivered to the pituitary was ≥50 Gy (HR 3.63, 95% CI [1.07; 12.30]). They remained significant in multivariate analysis. Age at diagnosis, gender and surgery before radiation therapy were not predictive of deficiency.

## Discussion

In this study of 52 patients with skull base meningiomas, the incidence of pituitary dysfunctions after cranial irradiation was high, up to 60.1% of patients at 10 years of treatment. We observed that hypopituitarism could appear very early within the first years after treatment, with increasing frequency thereafter.

Our study has good reproducibility, with a very homogenous population: regarding the radiation techniques, all the patients were treated using photon-beam therapy. Moreover, analyses for all 5 pituitary hormonal axes were available for all included patients, with a significant long follow-up (median follow-up was 7 years), which is a real strength in our study compared to others. Because of the presence of progesterone receptors in some meningiomas [10, 11], these tumors are mostly developed in women, which is the case in our population (80% of women).

In their study performed in 2005 on a general population of Northwestern Spain, Regal M. and al, [12] found a prevalence of 45.5/100000 of hypopituitarism, 61% of whom had pituitary tumors, which is very low compared to our prevalence of insufficiency. This supports the hypothesis of the role of cranial irradiation in the development of the observed pituitary hormonal axis deficiencies.

Previous studies showed that cranial radiotherapy almost systematically induces pituitary dysfunction with a range ranging from 25 to 100%, depending on the study considered [13–16]. One recent meta-analysis pooled 18 studies with a total of 813 patients who underwent cranial radiotherapy for head and nasopharynx tumors [17]. Amongst the patients, 31 had a meningioma. A total of 66% patients developed hypopituitarism as follows: 45% with GH deficiency, 30% with LH/FSH deficiency, 25% with TSH deficiency, 22% with ACTH deficiency and 34% with hyperprolactinemia. The important variation of prevalence between studies may be due to heterogeneous population, including cerebral and nasopharyngeal tumors with very different prognoses compared to meningiomas. It may also be due to a higher radiation dose exposure, or an incomplete endocrine evaluation in some studies (analysis including the 5 hormonal axes were not available in all patients).

As such, to the best of our knowledge, few studies focused specifically on the risk of cranial irradiation-induced hypopituitarism for meningioma of the skull base. Most previous studies have involved cerebral tumors and nasopharyngeal tumors. One recent study of 74 patients treated for sellar or parasellar meningiomas, with a median follow up of 3 years, found that 20% developed a pituitary dysfunction [18] which is in line with our result (19.2% at 3 years follow up) with thyrotroph and corticotroph axes being the most affected (24% each); the gonadotroph axis was the least affected with a prevalence at 10% and the somatotroph axis was affected in 19%. These differences may be explained by the fact that not all pituitary axes were studied for all patients (out of 74 patients, a somatotroph analysis was performed in only 16, and gonadotroph analysis in 31). Moreover, this group of patients was more heterogeneous than ours, with photon-beam therapy used in only 74% compared to all patients in our study.

Several studies have reported an increase in mortality due to hypopituitarism, underlining the need to screen patients following cranial irradiation [8].

Concerning the effect on the pituitary axes, the gonadotroph axis was the most affected in our study, with a prevalence of 36.9% at 10 years. 2.3% of these patients presented hyperprolactinemia.

Regal M and al [12] also found a higher prevalence for the gonadotroph axis among the axes affected, followed by the thyrotroph axis.

The least affected axis in our study was the somatotroph axis (13.4%), which differs in comparison to other studies. Indeed, in their meta-analysis Appelman and al [17]. observed GH deficiency in 45% of cases, which correlates well with the more frequent studies on children that show the somatotroph axis to be the most sensitive axis [19, 20]. Unlike adults, children are treated automatically regardless of initial signs of GH deficiency due to the important negative consequences on growth. Several hypotheses can be discussed to explain our findings of a lower prevalence of GH deficiency. First, in adults, GH cells are the most commonly found in the anterior pituitary gland and may explain why some loss of cells would go unnoticed, with GH production being compensated by the remaining cells [21]. Second, in contrast to other patient series, our group of patients was very homogeneous. All were treated for meningiomas, which have a very good prognosis, and overall the general status was better among our population, with lower rates of malnutrition and organ deficiencies. Levels of IGF1 correlate with nutrition status, hepatic and renal homeostasis, and insulin sensitivity [22, 23]. While the somatotroph axis appeared the least affected among our patients, analyses for most were carried out in an external laboratory with norms of GH and IGF1 that did not take age into consideration, and all patients were followed in out patient clinic in radiotherapy, which is not compatible with realization of the Growth Hormone Stimulation Test. New reference ranges used in laboratories as standard consider that pituitary hormone secretion is age-dependent [24]. One limitation of our study is the lack of any dynamic test for example the insulin test to screen for corticotroph and somatotroph deficiencies [25–27]. Such a test could be recommended in cases of discordance between clinical and laboratory tests or if adrenal analysis (cortisol) or IGF1 levels are low. Indeed, adrenal deficiency should be considered a real emergency. However, this dynamic test must be performed in a hospital setting and in the absence of contraindications (history of epilepsy, stroke and cardiac issues), due to the presence of unpleasant symptoms (in particular hypoglycemia), and therefore this test was contraindicated in several of our patients, at risk of seizures.

Due to a large panel of symptoms associated with their deficiencies, corticotroph, thyrotroph and gonadotroph axes represent the 3 most important axes requiring rapid treatment in the event of a deficiency. In contrast, somatotroph deficiency is barely compensated in adults as associated symptoms tend to be rare but mainly because such treatment increases the risk of tumor progression. In our study, as far as patients still presented meningioma residues, the safety concern of GH treatment remained unknown at the period of the study, and did not prompt clinicians to further investigate GH deficiency. Yet, all patients were treated for corticotroph and thyreotroph deficiency at diagnosis. Gonadotroph axis was not treated because of the age of the patients (> 50 years at diagnosis).

We also found predictive factors of hypopituitarism. Indeed, a dose to the pituitary gland ≥50 Gy or meningiomas measuring ≥40 mm were individually and significantly correlated with an increased risk of developing hypopituitarism. This concurs with earlier findings on skull base tumors by Pai et al. in 2001 [16], who found that a minimum dose to the pituitary ≥50 Gy significantly increased the risk of pituitary dysfunction. These authors also revealed a higher sensitivity to radiotherapy of the hypothalamus and recommended a maximum dose to this organ of 50 Gy. Concerning the dose, Littley et al. [14] in 1989 showed that hypopituitarism decreased with radiation doses < 20 Gy on the pituitary gland, whereas a dose between 35 and 45 Gy increased the risk. We are unable to confirm or not these earlier data with results from our study since a very high proportion of pituitary doses were ≥ 50 Gy (with a mean dose of 47 Gy and a range from15.9 to 54.5 Gy).

Interestingly, *P. Mehta and al* [28]. reported, in a retrospective study, the importance of sparing the hippocampus and the hypothalamus during a whole brain radiotherapy, using a volumetric modulated arc therapy. They showed that a simultaneous dose reduction to the hippocampus and the hypothalamus area did not compromise the planning target volume (PTV) coverage and thereby could be more used in routine.

Moreover, Pai et al. [16] also found that age > 40 years and histological type of tumor were individually predictive of pituitary dysfunction, especially along the corticotroph and thyrotroph axes. Concordantly, Regal et al. [12] found an 8 fold increase in risk of hypopituitarism among both sexes after 70 years of age, and a statistical difference between the age groups of 18–29 years and 30–49 years. We found no statistical differences in regard to these variables in our study, likely due to our patient group being highly homogenous with a mean age of 56.2 years +/− 14.1.

The precise pathophysiology behind this radiotherapy-induced deficiency remains unclear however one well-described hypothesis involves necrosis. The finding of increased sensitivity to irradiation among hypothalamic cells may lead to us shedding some light on the causative mechanisms [15, 29].

In agreement with that shown previously [16], our retrospective study has highlighted a correlation between cerebral irradiation for skull base meningioma and risk of hypopituitarism with a dose-dependence ≥50 Gy and a meningioma size ≥40 mm increasing this risk.

## Conclusions

To conclude, we observed a greater frequency of hypopituitarism among our patient population compared to the general population [12]. We also found that such deficiencies manifest differently according to which of the 5 hormonal axes is affected; this could guide physicians in their clinical examination of patients followed-up after radiotherapy. An annual follow up is recommended on all 5 axes for a minimum of 12 years; indeed GH deficiency still appears after 12 years of follow up.

We also found a correlation between the risk of hypopituitarism and a dose to the pituitary gland ≥50 Gy and meningiomas measuring ≥40 mm. These could represent predictive factors and alert physicians to the need for very assiduous monitoring in such patients. In light of these results, a maximum dose of 45 Gy should be applied whenever possible to reduce the toxicity to the pituitary gland. Both hypothalamus and pituitary gland critically require careful delineation in the CT planning stage of radiotherapy.

Nevertheless, prospective studies would be necessary to prove the causal relationship between these factors. It would also be interesting to compare photon-beam and proton therapies and the mean doses to both the pituitary gland and the hypothalamus, neither of which is at present routinely delineated in our center.

**Fig. 3:**
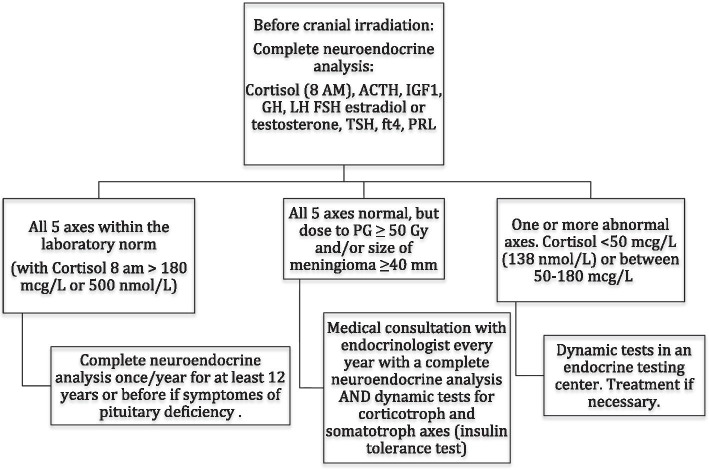
Decisional tree on recommanded follow up procedure according to neuroendocrine analysis results and predictive factors. Abbreviation: PG: pituitary gland.

Finally, the observed difference between prevalence in a general adult population and in our population, underlines the need for careful consideration of the risks to individual patients of hypopituitarism before cranial radiation therapy for skull base meningioma. We recommend adapting the course of action with regard to follow up procedure required according to the results of neuroendocrine analyses and predictive factors obtained before cranial radiation therapy, as proposed in our decisional tree (Fig. 3), in order to help radiotherapists in this specific circumstance.

## Data Availability

All data generated or analysed during this study are included in this published article [and its supplementary information files].

## Abbreviations

ACTH: Adrenocorticotrophic Hormone
BMI: Body Mass Index
CT: Computarized Tomography
CTV: Clinical Target Volume
FSH: Follicle-Stimulating Hormone
fT3: Free Tri-iodothyronine
fT4: Free Thyroxine
GH: Growth Hormone
GTV: Gross Tumor Volume
Gy: Gray
HR: Hazard Ratio
IGF1: Insulin Growth Factor 1
IMRT: Intensity Modulated Radiotherapy
LH: Luteinizing Hormone
MRI: Magnetic Resonance Imagery
PG: Pituitary Gland
PTV: Planning Target Volume
RT: Radiotherapy
TSH: Thyroid Stimulating Hormone
WHO: World Health Organization
